# Case Report: Nontuberculous mycobacterial infections in children with complete DiGeorge anomaly

**DOI:** 10.3389/fimmu.2023.1078976

**Published:** 2023-02-13

**Authors:** Elizabeth Daly Hicks, Noah O. Agada, Tyler R. Yates, Matthew S. Kelly, Jonathan S. Tam, Ronald M. Ferdman, Louis R. Dibernardo, John F. Madden, M. Anthony Moody, Mary Louise Markert

**Affiliations:** ^1^ Division of Pediatric Allergy, Immunology, and Pulmonology, Department of Pediatrics, Duke University Medical Center, Durham, NC, United States; ^2^ Division of Infectious Diseases, Department of Pediatrics, Duke University Medical Center, Durham, NC, United States; ^3^ Division of Clinical Immunology and Allergy, Children’s Hospital Los Angeles, Los Angeles, CA, United States; ^4^ Department of Pathology, Duke University Medical Center, Durham, NC, United States; ^5^ Department of Immunology, Duke University Medical Center, Durham, NC, United States

**Keywords:** athymia, complete DiGeorge syndrome, DiGeorge anomaly, nontuberculous mycobacteria, *Mycobacterium kansasii*, *Mycobacterium avium* complex, thymus transplantation, primary immunodeficiency

## Abstract

Children with complete DiGeorge anomaly (cDGA) have congenital athymia, resulting in severe T cell immunodeficiency and susceptibility to a broad range of infections. We report the clinical course, immunologic phenotypes, treatment, and outcomes of three cases of disseminated nontuberculous mycobacterial infections (NTM) in patients with cDGA who underwent cultured thymus tissue implantation (CTTI). Two patients were diagnosed with *Mycobacterium avium* complex (MAC) and one patient with *Mycobacterium kansasii*. All three patients required protracted therapy with multiple antimycobacterial agents. One patient, who was treated with steroids due to concern for immune reconstitution inflammatory syndrome (IRIS), died due to MAC infection. Two patients have completed therapy and are alive and well. T cell counts and cultured thymus tissue biopsies demonstrated good thymic function and thymopoiesis despite NTM infection. Based on our experience with these three patients, we recommend that providers strongly consider macrolide prophylaxis upon diagnosis of cDGA. We obtain mycobacterial blood cultures when cDGA patients have fevers without a localizing source. In cDGA patients with disseminated NTM, treatment should consist of at least two antimycobacterial medications and be provided in close consultation with an infectious diseases subspecialist. Therapy should be continued until T cell reconstitution is achieved.

## Introduction

DiGeorge anomaly (DGA) is a primary immunodeficiency associated with congenital defects of the heart, parathyroid glands, and thymus. Complete DGA (cDGA) is characterized by congenital athymia which often has fewer than 50 naive (thymically-derived) T cells/mm^3^ in peripheral blood (1–3). All patients with cDGA are susceptible to a broad range of infections secondary to the absence of thymically-derived T cells. Here we report the clinical presentation, treatment, and outcomes of nontuberculous mycobacterial (NTM) infection in three patients with cDGA who were evaluated for cultured thymus tissue implantation (CTTI) at Duke University Medical Center.

## Methods

All patients were enrolled in a research protocol approved by the Institutional Review Board at Duke University (ClinicalTrials.gov study NCT01220531). The parents provided written informed consent for their children to participate in the protocol (Pro00025966 Safety and Efficacy of Thymus Transplantation). The protocol was conducted under an Investigational New Drug (IND) Application with the Food and Drug Administration (FDA), IND#9836. The clinical, laboratory, and radiological data of all patients were reviewed. Naïve T cells were defined as CD3^+^ T cells expressing both CD45RA and CD62L.

## Results

**Figure 1:**
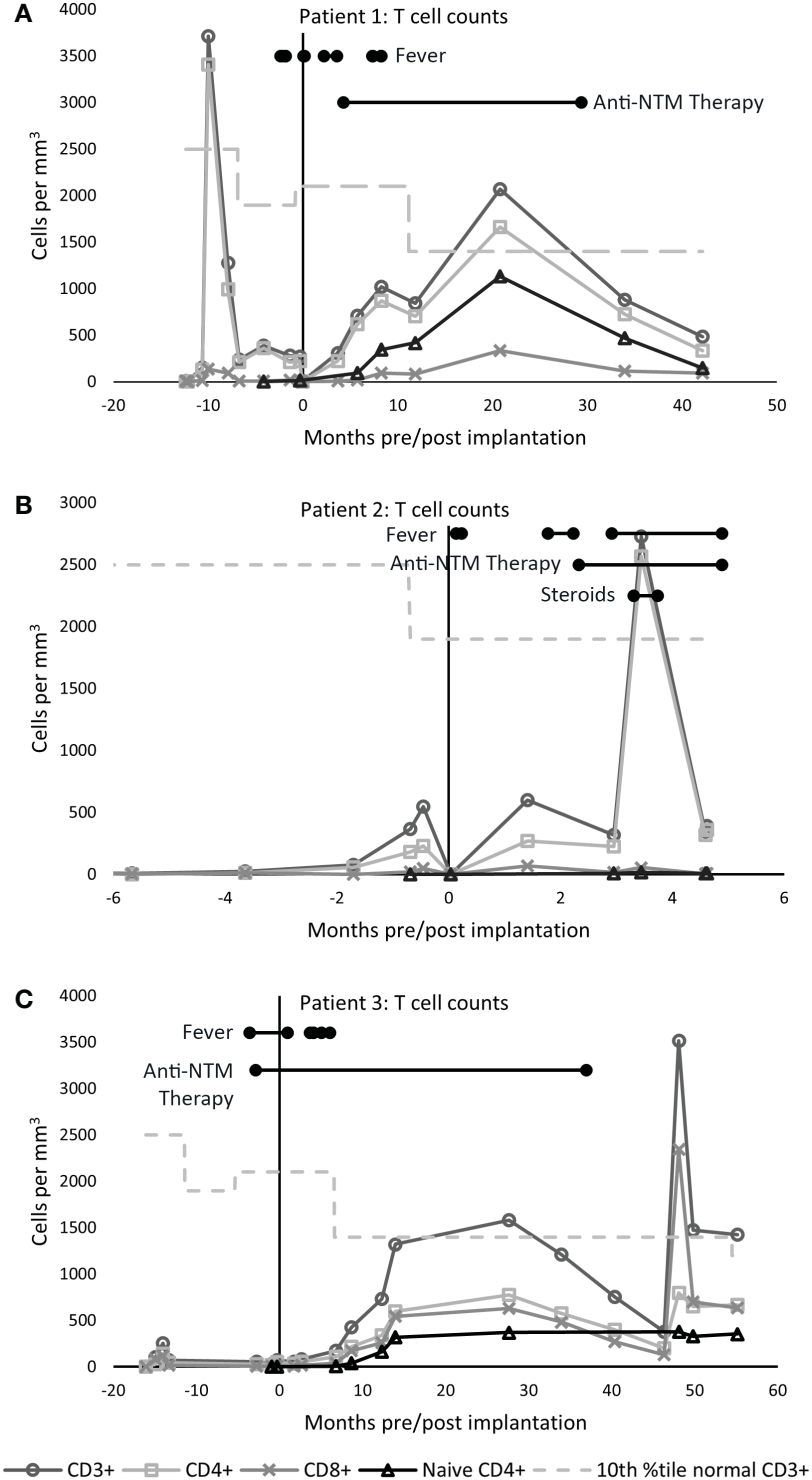
T cell counts. The tenth percentile of the normal CD3^+^ range for age is shown as a dashed line (4). **(A)** In patient 1, the CD3^+^ count increased to 3,710 cells/mm^3^ ten months prior to implantation, when the patient developed atypical cDGA. Naïve CD4^+^ T cells appeared at 5.7 months post-CTTI and increased steadily over the next several months. **(B)** Patient 2 had low, but detectable, naive T cells with 7 naïve CD4^+^ T cells/mm^3^ three months after transplant. The large increase in CD4^+^ T cells seen at day 105 post CTTI was concerning for development of immune reconstitution inflammatory syndrome. **(C)** In Patient 3, naïve T cells appeared 6.8 months after CTTI, and have persisted for over six years. The large increase in CD8^+^ cells at month 48 was due to acute Epstein-Barr virus infection.

Patient 1 was an infant of a diabetic mother. The newborn screening panel showed T cell receptor rearrangement excision circles (TRECs) of <200/mm^3^. Flow cytometry at two weeks of age was notable for lymphopenia with normal numbers of B lymphocytes and natural killer (NK) cells but absent naïve T lymphocytes (**Table 1**). No thymus tissue was identified on ultrasound of the left chest. This patient did not have a cardiac defect. Hypocalcemia was detected at day of life 18, at which point the infant was started on calcium supplementation. The diagnosis of cDGA was made at this time. Intravenous immunoglobulin replacement therapy and trimethoprim-sulfamethoxazole prophylaxis were initiated in the first 2 months of life. At 3 months of age, the patient developed diarrhea, an erythematous total body rash, alopecia, and eosinophilia. Histological examination of a skin biopsy demonstrated mild intercellular edema in the epidermis and a superficial perivascular lymphocytic infiltrate most of which consisted of CD3^+^CD4^+^ T cells. Flow cytometry at this time showed increased numbers of T cells, as can be seen in **Figure 1A**, 10 months pre-implantation. A molecular clonality analysis of the T cell receptor Vbeta genes detected T cell clones. The patient was diagnosed with atypical cDGA and started on cyclosporine as treatment for rash related to the oligoclonal T cells.

**Table 1:** Patient characteristics.

	Patient 1	Patient 2	Patient 3	Normal Values (4)
Underlying syndrome	Infant of a diabetic mother	CHARGE	22q11 deletion	
Comorbidities	Hypoparathyroidism	Complex congenital heart disease Colobomas Congenital hearing loss Vertebral anomalies Growth restriction	Ventricular septal defect Solitary kidney Velopharyngeal insufficiency	
Initial flow cytometry				
Age at time of assay	12 days	13 days	38 days	
CD3^+^ (cells/mm^3^)	9	14	0	2,500 – 5,500
CD4^+^ (cells/mm^3^)	2	0	0	1,600 – 4,000
CD8^+^ (cells/mm^3^)	8	12	0	560 – 1,700
Naive CD4+ cells (cells/mm^3^)	0		0	1,200 – 3,600
CD19^+^ (cells/mm^3^)	1439	507	2156	300 – 2,000
CD16^+^/CD56^+^ (cells/mm^3^)	906	519	1364	170 – 1,100
Pre-CTTI Proliferation Data				
Age at time of assay	347 days	28 days	508 days	
Phytohemagglutinin (cpm)	33,096	5,613	276	85,000 – 286,636*
Background (cpm)	589	NA	57	
CTTI				
Age at implantation	12.8 months	6.6 months	17.4 months	
Immunosuppression	Cyclosporine	Cyclosporine	None	
Pre-implantation Conditioning	Methylpred-nisolone Anti-thymocyte globulin	Methylpred-nisolone Anti-thymocyte globulin	None	

cpm: counts per minute.

NA: Not available.

*Normal range for Duke laboratory.

Consistent with the diagnosis of cDGA, patient 1 developed multiple infections in the first year of life including rotavirus gastroenteritis, *Escherichia coli* urinary tract infection, and *Enterococcus faecalis* wound and bloodstream infections. The patient was transferred to our facility for CTTI at eight months of age. After stabilization of patient 1 with gastrostomy tube placement and subsequent weight gain, the patient was given CTTI at 13 months of life with pre-CTTI methylprednisolone and rabbit anti-thymocyte globulin.

In the week following CTTI, the patient was intermittently febrile with a maximum temperature of 38.9°C. Although one blood culture grew *Candida albicans*, subsequent cultures did not, and computed tomography (CT) of the chest and abdomen showed no evidence for fungal disease. Fevers resolved after empiric fluconazole therapy. Two months after transplantation, the patient again had high fevers without localizing source. Blood cultures were negative, but serum β-D-glucan, a marker associated with fungal infection, was positive. Given concern for possible *Candida* infection, the patient was treated with intravenous fluconazole for one month, followed by oral fluconazole prophylaxis.

Despite fungal prophylaxis, one month later (3 months post CTTI), the patient was again admitted to the hospital for high fevers associated with cough and tachypnea. The patient had a newly enlarged right occipital lymph node. A chest CT was notable for mediastinal lymphadenopathy and right posterior apical lung consolidation. Over the ensuing ten days the patient continued to have high fevers despite broad-spectrum antibiotics. A repeat chest CT showed new airspace opacities. An open thoracotomy and lung biopsies of the right apex and right middle lobe revealed granulomatous lesions with numerous acid-fast bacilli. Mycobacterial cultures from the right middle lung nodule subsequently confirmed the presence of *Mycobacterium avium* complex (MAC). Antimycobacterial therapy was initiated with enteral clarithromycin, rifampin, and ethambutol.

The patient developed right middle lung lobe collapse five months after CTTI. Bronchoscopy found a hard, plastic-like plug of the right middle bronchus that was unable to be removed. This persisted despite aggressive chest physiotherapy and airway clearance measures. The patient ultimately required rigid bronchoscopy to remove the hard plug. Though largely asymptomatic, chest imaging continued to show multifocal airspace disease and right middle lobe consolidation. Two months later, the patient underwent left occipital lymph node excision and repeat bronchoscopy. Bronchoscopy revealed a sessile bronchial mass, which was removed by laser. Cultures from both the excised lymph node and the bronchial mass grew MAC.

While on antimycobacterial therapy, the patient had chronic diarrhea and poor weight gain. There was concern that the persistent mycobacterial disease was due to poor enteral absorption of medications. A central line was placed nine months post-CTTI for parenteral azithromycin, rifampin, ciprofloxacin, and amikacin. The patient was started on total parenteral nutrition. The patient then demonstrated consistent weight gain, resolution of diarrhea, and improvement in pulmonary disease. Nineteen months post-CTTI, the patient resumed enteral medical therapy with clarithromycin, rifampicin, and ethambutol. Clarithromycin was later changed to levofloxacin due to concern for ototoxicity.

By 30 months post-CTTI, chest imaging and physical exam had improved, and the patient had good T cell reconstitution and function (**Figure 1A**). Antimycobacterial therapy was discontinued after 25 months of therapy. The patient has been off mycobacterial medications for over 5 years with no sign of recurrence.

**Figure 2:**
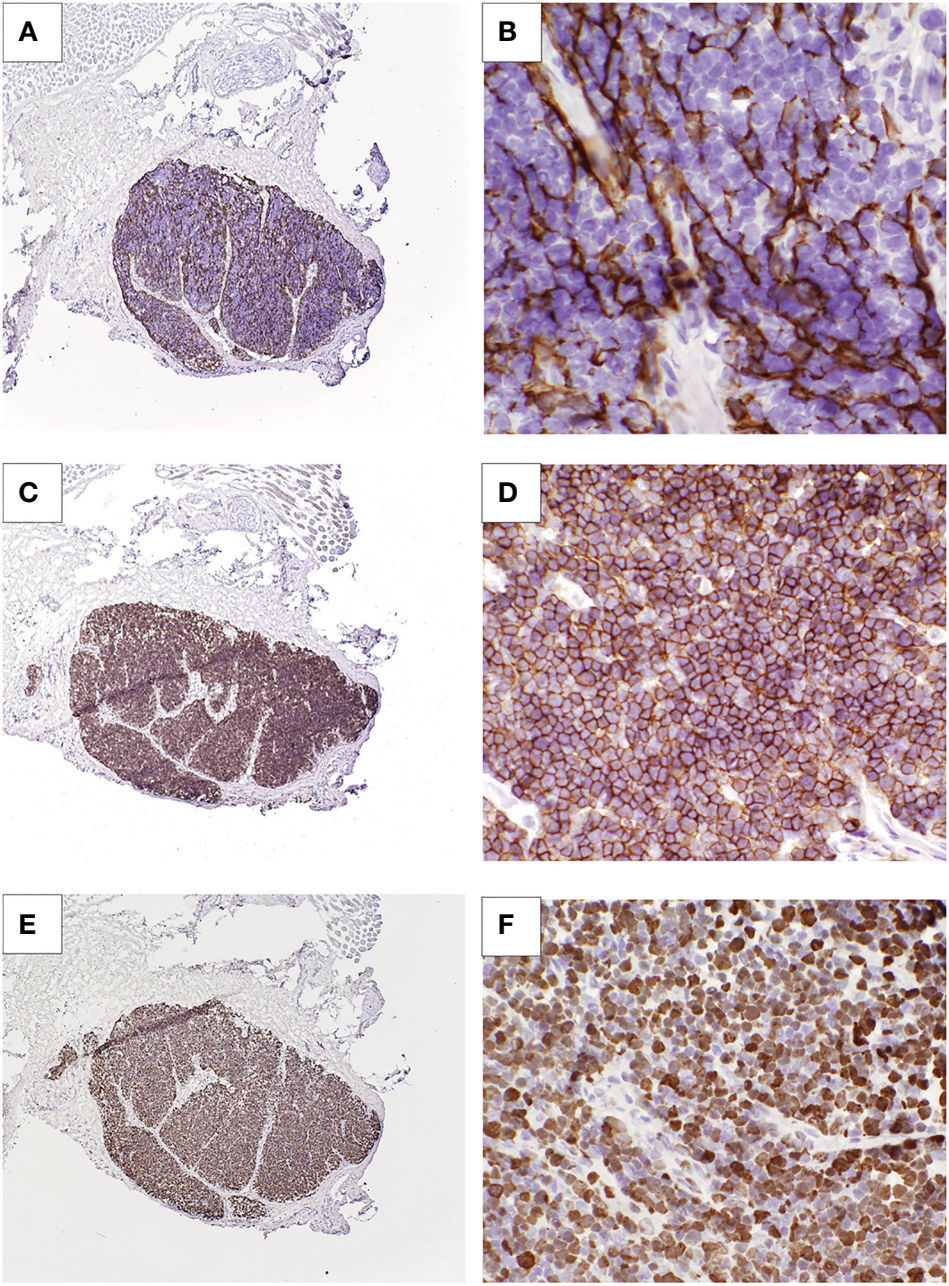
Cultured thymus tissue biopsy of patient 1. **(A, B)** Immunohistochemistry with anti-cytokeratin AE1/AE3 antibody shows thymic epithelial cells. **(C, D)** Anti-CD1a staining shows presence of thymocytes. **(E, F)** Staining for Ki-67, an intracellular marker for proliferating cells, show that these thymocytes are proliferating.

The protracted illness with *Mycobacterium avium* complex did not appear to hinder cultured thymus tissue engraftment. Biopsy of the cultured thymus tissue allograft 2 months after implantation showed thymopoiesis (**Figure 2**). Naïve T cells began to appear six months after implantation, and the naïve CD4 T cells were within the normal range by 21 months post-CTTI (**Figure 1A**). The patient received the live measles mumps rubella vaccine 4.5 years after CTTI, and the varicella vaccine six months later. Auditory brainstem response testing done during therapy showed mild high frequency hearing loss bilaterally, but this resolved on repeat testing 4 years after finishing therapy. Currently, the patient, although developmentally delayed, is growing well, and going to school.

Patient 2 was born with CHARGE syndrome (*c*oloboma, *h*eart defects, choanal *a*tresia, *r*etardation of growth or development, *g*enital hypoplasia, and *e*ar anomalies or deafness) with a premature stop mutation c.2371 C>T, Q791X, in chromo domain helicase DNA binding protein 7 (CHD7). The patient had congenital heart disease (bicuspid aortic valve, absent right superior vena cava, persistent left superior vena cava into the coronary sinus, mild aortic arch hypoplasia, and mild mitral valve stenosis), colobomas, growth restriction, genital hypoplasia, congenital hearing loss with malformed semicircular canals, and vertebral anomalies.

TRECs were undetectable on newborn screening. Flow cytometry was notable for lymphopenia with normal numbers of B cells and NK cell numbers, and very low T cells (**Table 1**). Lymphocyte proliferation studies showed very low responses to mitogen stimulation. These findings were consistent with a diagnosis of typical cDGA, associated with CHARGE syndrome.

The patient was started on trimethoprim-sulfamethoxazole prophylaxis and subcutaneous immunoglobulin replacement in the first month of life. Prior to implantation, the proliferation to phytohemagglutinin (PHA) stimulation increased to 21,372 counts per minute with a background of 301 counts per minute, which was concerning for development of atypical cDGA. Therefore, cyclosporine immunosuppression was initiated, and the patient received anti-thymocyte globulin and methylprednisolone prior to CTTI. The patient underwent CTTI at 6 months of age.

Four days after implantation, the patient developed a fever. Bacterial blood cultures were negative, and fevers resolved after seven days of cefepime and linezolid. No mycobacterial culture was obtained with this fever. Two months after CTTI, the patient was again admitted for intermittent high fevers without an apparent source. A body MRI showed thoracic and abdominal lymphadenopathy. Three mycobacterial blood cultures obtained during this admission grew *Mycobacterium avium* complex. Antimycobacterial therapy was begun with enteral clarithromycin, rifampin, and ethambutol.

**Figure 3:**
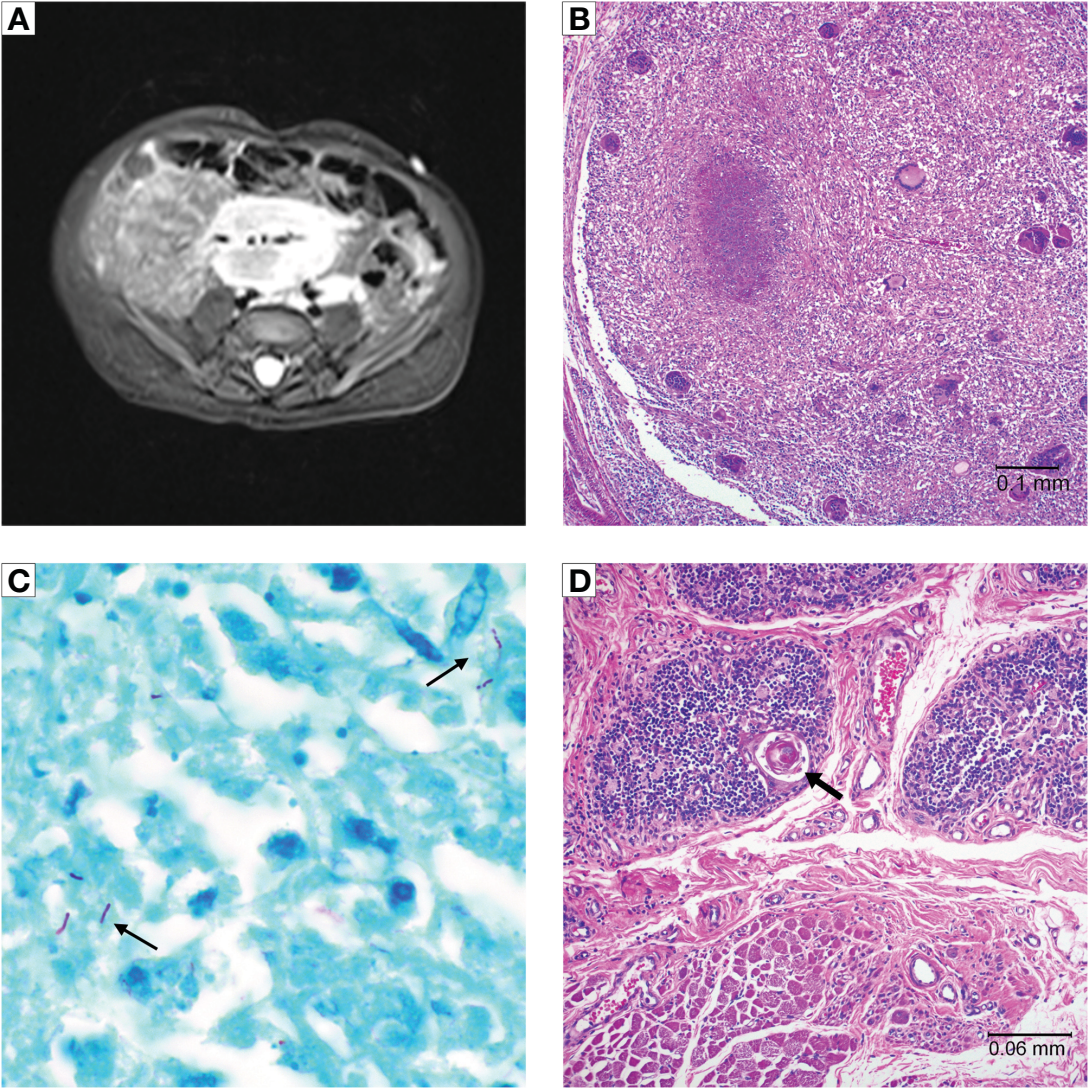
Patient 2 imaging and autopsy findings. **(A)** Abdominal MRI with lymphadenopathy surrounding mesenteric vessels. **(B)** H&E staining of a lymph node showing a caseating granuloma and giant cells. **(C)** Acid-fast staining of lymph node tissue showing presence of mycobacteria (arrows). **(D)** Cultured thymus tissue at time of autopsy. H&E staining shows intact Hassall bodies (arrow) surrounded by numerous thymocytes. The cultured thymus tissue graft was implanted in the quadriceps muscles. Muscle tissue is visible adjacent to the cultured thymus tissue.

The patient was admitted for recurrence of persistent high fevers 3 months after CTTI, along with intermittent tachypnea and feeding intolerance. An MRI showed a large nodal conglomeration within the lower abdomen surrounding the mesenteric vessels (**Figure 3A**), similar to the previous MRI. The large mass of lymph nodes raised the concern for lymphoproliferative disease, but it was too intertwined with the mesenteric vessels to allow for a biopsy. Blood polymerase chain reaction (PCR) for Epstein-Barr virus (EBV) was negative. The patient underwent biopsies of the bone marrow and liver to rule out EBV lymphoproliferative disease as cause for generalized lymphadenopathy, leukocytosis, and fevers. This evaluation was remarkable only for the presence of small non-caseating granulomas in both liver and bone marrow.

Flow cytometry showed new emergence of a small population of naïve T cells (3.3% of CD3^+^ cells), which in the context of continued fevers, tachypnea, and leukocytosis with underlying MAC infection, raised clinical suspicion for possible immune reconstitution inflammatory syndrome (IRIS). The patient was treated with 1 mg/kg/day of methylprednisolone for 8 days. Repeat flow cytometry 2 weeks later showed marked expansion of mature T cells from 214 CD4^+^ cells/mm^3^ to 2647 CD4^+^ cells/mm^3^, further suggesting that the patient’s symptoms could be related to IRIS (**Figure 1B**). The steroid dose was increased to 2 mg/kg/day for 5 days. However, the steroids did not lead to improvement in the clinical condition and were subsequently weaned off. The patient continued to deteriorate and developed respiratory failure and subsequent multi-organ failure four months after CTTI. Despite extensive investigations including multiple blood cultures and bronchoalveolar lavage (BAL) cultures, no etiology for the persistent fevers, lymphadenopathy, and respiratory distress was found aside from the disseminated mycobacterial infection. Patient 2 died 5 months after CTTI.

An autopsy revealed diffused adenopathy with granulomas (**Figure 3B**). Histologic exam of lymphatic tissue showed acid-fast bacilli, consistent with disseminated mycobacteria (**Figure 3C**). Examination of the implanted cultured thymus tissue showed normal architecture and thymocytes (**Figure 3D**).

Patient 3 was previously published in a case report by Yin, et al. (5), describing pre-CTTI findings. cDGA was suspected at birth based on absent TRECs on newborn screening plus the presence of a ventricular septal defect and solitary kidney. Additional immune studies performed at 5 weeks of age revealed lymphopenia with very low T cells, elevated NK cells, and elevated B cells (**Table 1**). Genetic studies revealed the presence of a chromosome 22q11.2 deletion. Genetic sequencing for SCID mutations was normal. Subsequent flow cytometry testing performed at 2 and 6 months of age again demonstrated very low T cells (**Figure 1C**). The parents declined treatment for immunodeficiency at this time.

At 13 months of age, patient 3 developed fever, cough, and difficulty feeding. Chest radiograph showed a right upper lobe lung opacity. PCR of BAL fluid revealed *Pneumocystis jirovecii* pneumonia (PJP). The patient was started on trimethoprim-sulfamethoxazole but continued to have fevers despite this treatment. CT scan showed significant lymphadenopathy in the chest and axillae and lytic lesions involving multiple osseous structures. A thoracoscopic lymph node biopsy showed numerous acid-fast bacilli, and biopsy culture grew *Mycobacterium kansasii*. Antimycobacterial therapy was started at 14 months of age with clarithromycin, rifabutin, and isoniazid.

The patient was referred to Duke University Medical Center where the patient underwent CTTI at 17 months of age with no pre- or post-transplant immunosuppressive medications. Patient 3 continued to have intermittent fevers. Despite extensive investigations including repeat BAL and a lung biopsy two weeks after CTTI, no alternative etiology for the fevers was identified. Due to worsening airspace disease seen on imaging and continued fever, antimycobacterial therapy was escalated to a four-drug regimen with clarithromycin, rifampin, isoniazid, and moxifloxacin along with three weeks of intravenous linezolid. Interferon gamma infusions were started three times weekly. The patient defervesced and began to improve on this regimen. High fevers returned two months later (4 months post-CTTI), which were treated with four weeks of IV linezolid and IV amikacin in addition to his other antimycobacterial medications. His disease remained well controlled on oral medications after this.

Flow cytometry 1 year post-CTTI revealed development of naïve T cells (**Figure 1C**). Patient 3 remained on anti-mycobacterial therapy until 37 months post-CTTI. The patient tolerated live varicella and measles, mumps, rubella vaccines 5 years after CTTI. Patient 3 is now 6.5 years post-CTTI and has been off all antimycobacterial therapy for almost 4 years with no signs of recurrence of *Mycobacteria*. Hearing evaluation 1 year after stopping therapy showed mild hearing loss at the highest tested frequency that was considered clinically insignificant. The patient is going to school and participating fully in all activities.

## Discussion

This report presents three patients with cDGA who developed non-tuberculous mycobacterial infections. They encompassed the three most common categories of patients with cDGA: infants of diabetic mothers (patient 1), CHARGE syndrome (patient 2), and 22q11.2 deletions (patient 3) (6, 7). None had naïve T cells prior to CTTI. Patient 1 had atypical cDGA with a presentation similar to Omenn syndrome and a high T cell count prior to starting immunosuppression. In addition to athymia, patients 1 and 2 had hypoparathyroidism, and patients 2 and 3 had cardiac defects. Patients 1 and 3 survived, and patient 2 died.

These cases demonstrate multiple challenges in diagnosing and treating mycobacterial infections in patients with T cell immune deficiencies. Our patients highlight how the insidious, nonspecific nature of mycobacterial infection makes timely diagnosis difficult. All three patients had multiple admissions and evaluations for fevers prior to discovering mycobacterial infection. Patient 1’s initial fevers were attributed to *Candida*, but in retrospect, may have been due to NTM infection. Tissue biopsies were required for diagnosis in two of the patients.

Disseminated NTM was previously thought to be rare in patients with primary T cell immunodeficiencies (8). There are very few reports of NTM in severe combined immunodeficiency (SCID) or DiGeorge anomaly (9, 10). Most experience with disseminated non-tuberculous mycobacterial infections comes from patients with advanced human immunodeficiency virus (HIV)/acquired immunodeficiency syndrome (AIDS) (11, 12). In HIV/AIDS, disseminated NTM is considered a late finding. Most children with HIV have had profound T cell lymphopenia (CD4 counts less than 50 cells/mm^3^) for over one year prior to development of disseminated NTM (11, 12). With three cases out of approximately 100 cDGA patients treated at Duke, it is not clear why disseminated NTM appears to be more frequent in our cohort of cDGA patients than in SCID or other severe T cell immunodeficiencies. One possible factor is the length of time until intervention. The median age of bone marrow transplantation for babies with SCID is 103 days (13), compared to a median age at CTTI of 298 days in cDGA patients (Markert, unpublished data). Many patients with cDGA require stabilization and treatment of associated congenital anomalies prior to CTTI. This prolonged period of T cell lymphopenia prior to CTTI may increase the risk of disseminated NTM in these patients.

Similar to children with HIV/AIDS, our patients received aggressive and protracted treatment with three or more antimicrobials for over two years. Patient 1 was treated with parental therapy for several months. Patient 2 succumbed to disease despite multi-drug therapy. Patient 3 received a regimen of five anti-mycobacterial medications in addition to interferon-gamma therapy before demonstrating clinical improvement.

Patient 2 was treated for possible IRIS with cyclosporine and steroids after recurrence of fevers despite anti-mycobacterial therapy. This patient had an associated 10-fold increase in peripheral T lymphocytes on flow cytometry that was initially considered to be indicative of T cell recovery (**Figure 1B**). We now think that IRIS is unlikely to occur in athymic patients in the first few months after CTTI as the naïve T cell counts are still very low and may not be able to generate sufficient effector cells to cause IRIS. This is in contrast to HIV/AIDS, where NTM is a known cause of IRIS in patients starting antiretroviral therapy (14). Further, use of steroids in cDGA patients may hinder thymopoiesis, delaying T cell reconstitution, which is needed for clearance of NTM (15). Based on our experience with patient 2 and discussions with other centers, we try to avoid use of steroids in cDGA patients with mycobacterial infections. However, not enough is known about the risk for IRIS in these patients to make strong recommendations on the use of steroids.

There are several recommendations that we would make based on our experience with these patients. When patients are first diagnosed with cDGA, we recommend prophylaxis with azithromycin or clarithromycin. This is based on the IDSA guidelines for children with advanced HIV/AIDS and on randomized clinical trials in HIV/AIDS patients with less than 100 T cells/mm^3^ that have shown reduced rates of NTM infection with macrolide prophylaxis (16, 17). If there is any concern for pre-existing mycobacterial infection, strongly consider obtaining a screening mycobacterial culture prior to initiating prophylaxis. Closer monitoring and dose adjustments may be necessary in patients on calcineurin inhibitors due to drug interactions with the macrolide antibiotic resulting in higher levels of the calcineurin inhibitor. We recommend obtaining mycobacterial blood cultures whenever the patient has a fever without a localizing source. Unfortunately, in this small number of patients, inflammatory markers such as C-reactive protein and ferritin were not helpful in diagnosis. Complete DGA patients with disseminated NTM may require treatment with at least two antimycobacterial medications for at least 12 months. This should be done in consultation with an infectious diseases expert. Therapy should be continued until the patient has good T cell reconstitution.

We were pleased to find that mycobacterial infection did not destroy the thymic allograft in these patients. Patient 1 had thymopoiesis on the allograft biopsy obtained 2 months after CTTI (**Figure 2**). Eight years after CTTI, this patient continues to have good thymic output as indicated by presence of naïve T cells. Patient 2 had evidence of thymopoiesis at autopsy (**Figure 3D**) and low but detectable circulating naïve T cells at 3 months after CTTI. Patient 3 also has sustained thymic output (**Figure 1C**).

## Conclusions

A diagnosis of NTM infection should be considered in all patients with cDGA or other T cell deficiencies who present with fever, lymphadenopathy, or respiratory symptoms. Aggressive and prolonged anti-mycobacterial therapy is recommended, and steroids should generally be avoided in CTTI patients. We recommend that children with cDGA receive macrolide prophylaxis until T cell reconstitution occurs. Increased awareness of the possibility of NTM infection in cDGA will lead to earlier consideration of this diagnosis.

## Data availability statement

The datasets generated during and/or analyzed during the current study are available from the corresponding author on reasonable request. Requests to access these datasets should be directed to Elizabeth Hicks, edh19@duke.edu.

## Ethics statement

The studies involving human participants were reviewed and approved by Duke University Institutional Review Board under the IRB protocol Pro00025966, Safety and Efficacy of Thymus Transplantation. Written informed consent to participate in this study was provided by the participants’ legal guardian/next of kin.

## Author contributions

EDH, NOA, TRY, MSK, MLM and MAM contributed to writing the manuscript. JT and RF referred patient 3 and provided clinical information. LD and JM provided histology and autopsy findings. All authors contributed to the article and approved the submitted version.

## References

[1] Markert ML Sarzotti M Ozaki DA Sempowski GD Rhein ME Hale LP. Thymus transplantation in complete DiGeorge syndrome: Immunologic and safety evaluations in 12 patients. Blood (2003) 102(3):1121–30. doi: 10.1182/blood-2002-08-2545 12702512

[2] Markert ML Devlin BH Alexieff MJ Li J McCarthy EA Gupton SE. Review of 54 patients with complete DiGeorge anomaly enrolled in protocols for thymus transplantation: Outcome of 44 consecutive transplants. Blood (2007) 109(10):4539–47. doi: 10.1182/blood-2006-10-048652 PMC1885498 17284531

[3] Markert ML Devlin BH McCarthy EA. Thymus transplantation. Clin Immunol (2010) 135(2):236–46. doi: 10.1016/j.clim.2010.02.007 PMC3646264 20236866

[4] Shearer WT Rosenblatt HM Gelman RS Oyomopito R Plaeger S Stiehm ER. Lymphocyte subsets in healthy children from birth through 18 years of age: The pediatric AIDS clinical trials group P1009 study. J Allergy Clin Immunol (2003) 112(5):973–80. doi: 10.1016/j.jaci.2003.07.003 14610491

[5] Yin SM Ferdman RM Wang L Markert ML Tam JS. Disseminated mycobacterium kansasii disease in complete DiGeorge syndrome. J Clin Immunol (2015) 35(5):435–8. doi: 10.1007/s10875-015-0171-3 26048260

[6] de Lonlay-Debeney P Cormier-Daire V Amiel J Abadie V Odent S Paupe A. Features of DiGeorge syndrome and CHARGE association in five patients. J Med Genet (1997) 34(12):986–9. doi: 10.1136/jmg.34.12.986 PMC1051148 9429139

[7] Wang R Martinez-Frias ML Graham JM Jr. Infants of diabetic mothers are at increased risk for the oculo-auriculo-vertebral sequence: A case-based and case-control approach. J Pediatr (2002) 141(5):611–7. doi: 10.1067/mpd.2002.128891 12410187

[8] Wu UI Holland SM. Host susceptibility to non-tuberculous mycobacterial infections. Lancet Infect Dis (2015) 15(8):968–80. doi: 10.1016/S1473-3099(15)00089-4 26049967

[9] Hoyos-Bachiloglu R Gallo S Vizcaya C Zuniga P Valbuena JR Casanova JL. Disseminated mycobacterial disease in a patient with 22q11.2 deletion syndrome: Case report and review of the literature. J Clin Immunol (2019) 39(7):743–6. doi: 10.1007/s10875-019-00678-5 PMC7159191 31385124

[10] Parent LJ Salam MM Appelbaum PC Dossett JH. Disseminated mycobacterium marinum infection and bacteremia in a child with severe combined immunodeficiency. Clin Infect Dis (1995) 21(5):1325–7. doi: 10.1093/clinids/21.5.1325 8589169

[11] Horsburgh CR Jr. Caldwell MB Simonds RJ. Epidemiology of disseminated nontuberculous mycobacterial disease in children with acquired immunodeficiency syndrome. Pediatr Infect Dis J (1993) 12(3):219–22. doi: 10.1097/00006454-199303000-00009 8095716

[12] Hoyt L Oleske J Holland B Connor E. Nontuberculous mycobacteria in children with acquired immunodeficiency syndrome. Pediatr Infect Dis J (1992) 11(5):354–60. doi: 10.1097/00006454-199205000-00003 1630855

[13] Heimall J Logan BR Cowan MJ Notarangelo LD Griffith LM Puck JM. Immune reconstitution and survival of 100 SCID patients post-hematopoietic cell transplant: a PIDTC natural history study. Blood (2017) 130(25):2718–27. doi: 10.1182/blood-2017-05-781849 PMC5746165 29021228

[14] Puthanakit T Oberdorfer P Ukarapol N Akarathum N Punjaisee S Sirisanthana T. Immune reconstitution syndrome from nontuberculous mycobacterial infection after initiation of antiretroviral therapy in children with HIV infection. Pediatr Infect Dis J (2006) 25(7):645–8. doi: 10.1097/01.inf.0000225786.00940.37 PMC1924531 16804438

[15] Ashwell JD Lu FW Vacchio MS. Glucocorticoids in T cell development and function. Annu Rev Immunol (2000) 18:309–45. doi: 10.1146/annurev.immunol.18.1.309 10837061

[16] Children PoOIiH-EaH-I. guidelines for the prevention and treatment of opportunistic infections in HIV-exposed and HIV-infected children. Available at: https://clinicalinfo.hiv.gov/en/guidelines/pediatric-opportunistic-infection.

[17] Oldfield EC 3rd Fessel WJ Dunne MW Dickinson G Wallace MR Byrne W. Once weekly azithromycin therapy for prevention of mycobacterium avium complex infection in patients with AIDS: A randomized, double-blind, placebo-controlled multicenter trial. Clin Infect Dis (1998) 26(3):611–9. doi: 10.1086/514566 9524832

